# Knowledge of Diabetic Complications in Diabetic Patients and Non-diabetic Family Care Givers: An Awareness of Health Promotion Study

**DOI:** 10.7759/cureus.96175

**Published:** 2025-11-05

**Authors:** Jayshree Dawane, Chandra D Shukla, Pradumna Shinde

**Affiliations:** 1 Pharmacology, Bharati Vidyapeeth (Deemed to be University) Medical College, Pune, Pune, IND

**Keywords:** awareness, diabetic complications, family caregivers, health promotion, patient education

## Abstract

Background

Diabetes mellitus and its complications have a significant impact on patient health and quality of life as they impose substantial burdens on family members. This study aims to explore the awareness levels of diabetic complications among both diabetic patients and their non-diabetic family caregivers.

Objectives

To assess the level of knowledge and its association with sociodemographic factors concerning diabetes management and complications among diabetic patients and their caregivers.

Methodology

A cross-sectional, questionnaire-based descriptive study was conducted in the Diabetes Specialty Clinic of a tertiary health care hospital in Pune. The study included diabetic patients attending the OPD as well as inpatients, along with their caregivers. For patients who were not accompanied by their caregivers, information was gathered via telephone. A total of 65 diabetic patients and 65 caregivers were included in the study. Section A of the questionnaire collects demographic information and disease-related information. Section B assessed basic knowledge of diabetic complications, chronic co-morbidities, foot care, and peripheral neuropathy. Participants were also asked about their apprehensions regarding diabetic complications. Data were analysed using SPSS Statistics version 21 (IBM Corp., Armonk, NY, USA).

Results

The majority of patients and caregivers demonstrated limited awareness of complications associated with sustained hyperglycemia. Only 40 patients (61.5%) and 31 caregivers (47.6%) recognized myocardial infarction (MI) as a possible outcome. Awareness of stroke was similarly low, with 33 patients (50.7%) and eight caregivers (12.3%) identifying it as a risk. Recognition of congestive cardiac failure (CCF) was poorest, with just 30 patients (46.2%) and nine caregivers (13.8%) aware of its link to prolonged high blood sugar levels. Patients had higher knowledge scores compared to caregivers, indicating a need for targeted educational interventions for family caregivers especially towards monitoring regular sugar, adherence to treatment and lifestyle modification.

Conclusion

The results highlight significant gaps in knowledge and underscore the influence of socio-demographic factors on diabetes management and awareness. These findings can inform the development of targeted health promotion strategies aimed at improving diabetes care and preventing complications.

## Introduction

Diabetes mellitus is a chronic metabolic disorder characterized by hyperglycemia, resulting from defects in insulin secretion, insulin action, or both. It is a significant public health concern worldwide, contributing to substantial morbidity, mortality, and healthcare costs [1].

Diabetes mellitus affects approximately 463 million adults (aged 20-79 years) worldwide [2]. Type 2 diabetes is the most common form, accounting for about 90% of cases. India has one of the highest burdens of diabetes globally. According to the International Diabetes Federation (IDF), approximately 77 million people in India had diabetes in 2019 [3]. Effective management of diabetes involves not only pharmacological interventions but also comprehensive education about lifestyle modifications and disease monitoring for the prevention of complications [4].

Diabetic complications can broadly be categorized into microvascular and macrovascular issues. Microvascular complications involve damage to small blood vessels, leading to diabetic retinopathy affecting the eyes, nephropathy affecting the kidneys, and neuropathy affecting nerves throughout the body [5]. On the other hand, macrovascular complications affect larger blood vessels, increasing the risk of cardiovascular diseases, stroke, and peripheral vascular disease [6]. Effective management of diabetes involves maintaining optimal blood glucose levels, controlling blood pressure and cholesterol levels and regular monitoring to detect and mitigate the onset and progression of these complications, thereby improving overall quality of life and reducing the burden of disease. Complications have a significant impact on patients' quality of life [7] and increase the burden on healthcare systems [8]. Awareness and knowledge of these complications are crucial for both diabetic patients and their caregivers to ensure timely intervention and optimal management [9]. Early detection and intervention are crucial in preventing or slowing the progression of diabetic complications [10].

Family caregivers play a vital role in the care and support of diabetic patients, particularly in managing daily routines, medication adherence, and lifestyle changes [11]. Their understanding of the disease and its complications is essential for providing effective care and preventing adverse outcomes [12]. However, there is often a gap in knowledge between patients and their caregivers, which can hinder effective diabetes management.

This study aims to evaluate the extent of knowledge about diabetes management and its complications among diabetic patients and their non-diabetic family caregivers at a diabetes specialty clinic of a tertiary care centre, and how this knowledge varies across sociodemographic factors. Through the assessment of patients’ and caregivers’ knowledge, the study seeks to highlight priority areas for focused educational strategies. The findings will inform health promotion strategies aimed at enhancing diabetes management and reducing the incidence of complications.

## Materials and methods

A cross-sectional, questionnaire-based descriptive study was conducted in the Diabetes Specialty Clinic of a tertiary health care hospital, Bharati Vidyapeeth Deemed University Medical College and Hospital, Pune. Before starting the study, approval from the Institutional Ethical Committee was obtained (approval BVDUMC/IEC/22). A total of 130 participants were recruited through convenience sampling, comprising 65 diabetic patients attending the outpatient department or admitted as inpatients, along with their respective 65 non-diabetic family caregivers. Informed consent was obtained from all participants prior to data collection. For patients who were not accompanied by caregivers, caregiver information was obtained telephonically. Data collection was carried out over a three-month period, from April 1, 2024, to June 30, 2024.

Data were collected from patients and caregivers, including those visiting the outpatient department and those contacted telephonically, using a predesigned, structured, and validated questionnaire. The questionnaire underwent validation through pilot testing and expert review by the Faculty of the Department of Pharmacology. The answer to each question was reviewed by experts and the requisite modifications and deletions were made. An explanatory statement was provided to all participants, detailing the purpose of the study, assurance of confidentiality of their responses, information regarding consent and right to withdraw at any stage, as well as addressing any questions or concerns they had regarding the study. The questionnaire was divided into two sections. Section A comprised 25 questions for the collection of demographic information and disease-related data, including patient age, race, residence, years since diagnosis, type of diabetic medication, current diabetes status and multiple-choice questions covering key aspects of diabetes management such as hypoglycaemic symptom identification, plasma glucose level awareness, and knowledge of diet and exercise. Section B comprised 32 questions; Part A had 18 questions for the assessment of basic knowledge of diabetic complications, potential chronic co-morbidities, foot care, and peripheral neurological issues while Part B, with 14 questions, was for self-care parameter knowledge. For both Part A and Part B, responses were analysed descriptively. Each item was evaluated based on the frequency and percentage of participants selecting each option. The data were presented as percentage to reflect the overall distribution and trends in participants' knowledge and self-care practices related to diabetes and its complications. Participants were encouraged to seek clarifications from the interviewer about any concerns they had related to diabetic complications. The interviewers were adequately trained and provided with detailed knowledge about diabetes and its associated complications to ensure accurate communication and consistency in data collection.

Inclusion criteria

Diabetic patients and family caregivers attending the medicine outpatient as well as inpatient departments who signed the consent form.

Exclusion criteria

Patients who were excluded from the study included diabetic patients and family caregivers attending medicine outpatient and inpatient departments who did not provide signed consent. In addition, any questionnaires containing incomplete information or missing data were omitted from the final analysis. 

Statistical analysis

Data analysis was performed using the SPSS Statistics version 21 (IBM Corp., Armonk, NY, USA). Qualitative data were summarized using frequencies and percentages to describe participants' and caregivers' characteristics and knowledge levels. To examine the relationship between sociodemographic characteristics and knowledge, a one-way analysis of variance (ANOVA) was conducted using GraphPad Prism version 6 (La Jolla, CA, USA). Additionally, Pearson’s partial correlation coefficient was applied to assess the associations among income, residence and knowledge related to diabetes management and complications. All statistical tests were conducted with significance levels set at p < 0.05.

Data availability

Data used for this study are available from the corresponding author on request.

## Results

The study comprised a total of 130 participants, evenly divided between 65 diabetic patients and their 65 non-diabetic family caregivers. All participants completed the questionnaire, with no exclusions due to incomplete responses.

Demographic characteristics of participants

Table 1 presents the demographic characteristics of 65 diabetic patients and 65 non-diabetic caregivers, showing both numbers and percentages for each category. Among patients, the majority were aged 51-65 years (32 participants, 49.2%), followed by 65 and above (13, 20.0%), 36-50 years (14, 21.5%), and the smallest group, 20-35 years (6, 9.2%). Caregivers, in contrast, were generally younger, with the highest group being 36-50 years (20, 30.7%) and 20-35 years (15, 23%), while only 18 (27.7%) were 51-65 years and 12 (18.4%) were 65 or older. Regarding gender, most patients were female (44, 67.7%) and most caregivers were male (42, 64.6%). Examining educational status, primary school was completed by 23 patients (35.3%) and 10 caregivers (15.3%). Secondary education was more common among caregivers (40, 61.5%) than patients (25, 38.4%). Graduation was attained by 17 patients (26.1%) and 15 caregivers (23.0%).

**Table 1:** Demographic characteristics of participants

Demographic Variable		Patients Number(%)	Caregivers Number(%)
Age (years)	20-35	6(9.2)	15(23)
	36-50	14(21.5)	20(30.7)
	51-65	32(49.2)	18(27.7)
	65 and +	13(20.0)	12(18.4)
Gender	Male	21(32.3)	42(64.6 )
	Female	44(67.7)	23(35.3)
Educational Status	Primary	23(35.3)	10(15.3)
	Secondary	25(38.4)	40(61.5)
	Graduation	17(26.1)	15(23.0)
Residence	Urban	21(32.3)	21(32.3)
	Rural	44(67.6)	44(67.7)
Duration of Diabetes Mellitus (years)	0-5	27(41 .5)	NA
	6-10	19(29.2)	NA
	11-14	9(13.8)	NA
	15 and +	10(15.3)	NA

In terms of residence, a majority in both groups lived in rural areas: 44 patients (67.6%) and 44 caregivers (67.7%), with only 21 in each group (32.3%) residing in urban environments. For the duration of diabetes among patients, 27 (41.5%) had diabetes for 0 to five years, 19 (29.2%) for six to 10 years, nine (13.8%) for 11 to 15 years, and 10 (15.3%) for more than 15 years.

This demographic breakdown highlights that diabetic patients in this study tended to be older and female, with many having only primary or secondary education, and most living in rural settings. In contrast, caregivers skewed younger and male, with secondary education being most common, and also predominantly came from rural backgrounds. This context is essential for interpreting subsequent findings on diabetes awareness and self-care practices in the study population.

Knowledge about self-care in diabetes mellitus

**Figure 1:**
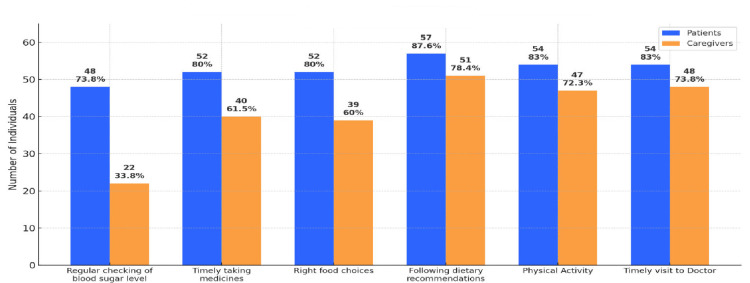
Knowledge about self-care in diabetes mellitus

Figure 1 depicts a bar graph comparing self-care knowledge in diabetes mellitus between patients and their caregivers, presenting both the number of individuals and corresponding percentages for each self-care domain.

In regular checking of blood sugar levels, 48 patients (73.8%) demonstrated knowledge or practice compared to only 22 caregivers (33.8%), highlighting a substantial gap in this crucial area. For timely taking of medicines, 52 patients (80%) reported good knowledge or practice, whereas this was true for 40 caregivers (61.5%). Right food choices were acknowledged by 52 patients (80%) and 39 caregivers (60%). The gap narrows in following dietary recommendations, with 57 patients (87.6%) and 51 caregivers (78.4%) indicating good knowledge. For physical activity, 54 patients (83%) recognized its importance compared to 47 caregivers (72.3%). Timely visits to the doctor were reported by 54 patients (83%) and 48 caregivers (73.8%).

Overall, the results demonstrate that diabetic patients consistently possess greater knowledge and adherence to self-care practices compared to their caregivers, with the largest differences seen in blood sugar monitoring and medication adherence. Caregiver knowledge, while reasonable in areas like diet, activity, and doctor visits, lags behind that of patients in every domain, underlining the need for targeted education and support to empower caregivers as effective partners in diabetes management.

Knowledge about chronic complications of diabetes mellitus

**Figure 2:**
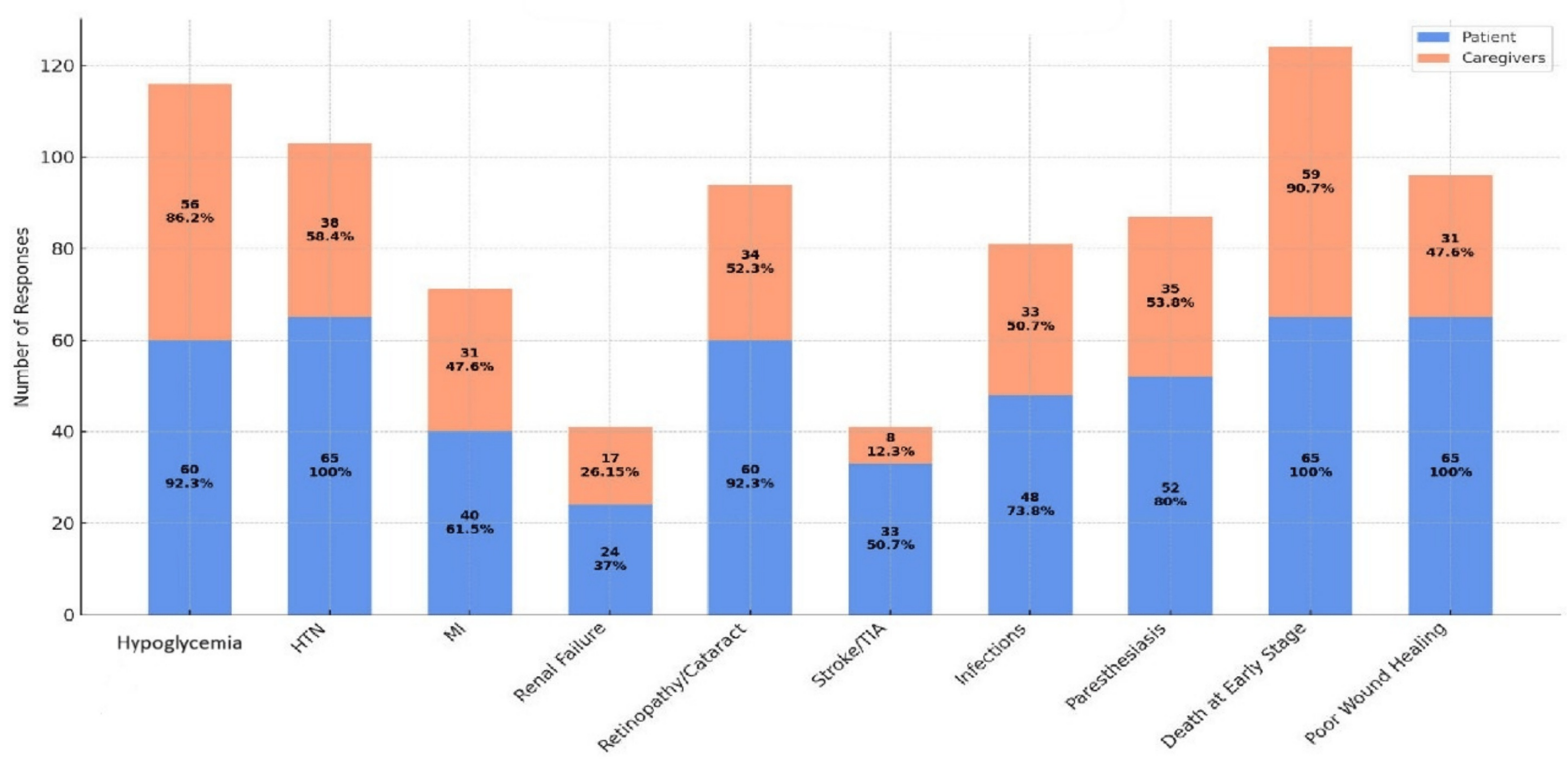
Knowledge about chronic complications of diabetes mellitus

The bar graph in Figure 2 displays how well diabetic patients and their caregivers recognize various chronic complications of diabetes mellitus, presenting both the number and percentage of participants aware of each condition. Hypoglycemia was identified as a complication by 60 patients (92.3%) and 56 caregivers (86.2%), showing high awareness among both groups. All patients (65, 100%) were aware of hypertension (HTN), but only 38 caregivers (58.4%) recognized it. Knowledge of myocardial infarction (MI) was moderate, with 40 patients (61.5%) and 31 caregivers (47.6%) indicating awareness.

A marked knowledge gap appeared for renal failure, with just 24 patients (37%) and 17 caregivers (26.15%) identifying it as a complication. Awareness of retinopathy or cataract was high among patients (60, 92.3%) but considerably less among caregivers (34, 52.3%). Stroke or transient ischemic attack (TIA) was recognized by 33 patients (50.7%) but only by eight caregivers (12.3%), highlighting a substantial caregiver deficit. Awareness of infections was reported by 48 patients (73.8%) and 33 caregivers (50.7%). For paraesthesias, 52 patients (80%) and 35 caregivers (53.8%) were knowledgeable. The awareness of the possibility of death at an early stage due to diabetes complications was very high, with all patients (65, 100%) and 59 caregivers (90.7%) recognizing it. Poor wound healing was known by all patients (65, 100%) but only 31 caregivers (47.6%).

Overall, patients were consistently more knowledgeable than caregivers across all chronic complications measured, with the most pronounced differences seen in conditions such as stroke/TIA, poor wound healing, and retinopathy/cataract. 

Association of knowledge with socio-demographic factors

Table 2 demonstrates the association between knowledge of diabetes complications and socio-demographic factors among patients and caregivers, providing both number and percentage for each category.

**Table 2:** Association of knowledge with socio-demographic factors Pearson Correlation Coefficient: Measures how strongly the parameter correlates with knowledge (range: -1 to +1). A higher positive value means a stronger relationship.

Parameter		Knowledge Of Patient	Knowledge of Caregiver	Pearson Correlation Coeffecient
Income	<5000	208(54.02)	177(45.98)	0.4132
	5000- 10000	288(55.70)	229(44.30)	NA
	>10000	339(57.3)	258(42.70)	NA
Education	School	230(67.13)	134(46.79)	0.736
	University	523(70.98)	453(58.18)	NA
Residence	Rural	330(57.13)	214(36.18)	0.423
	Urban	480(67.98)	253(44.20)	NA

For income, patients earning less than 5,000 had a knowledge score of 208 (54.02%), those earning 5,000-10,000 had 288 (55.7%), and those with income above 10,000 had 339 (57.3%). Corresponding figures for caregivers were 177 (45.98%), 229 (44.3%), and 258 (42.7%), respectively. The Pearson correlation coefficient of 0.4132 indicates a moderate positive correlation between higher income and greater knowledge. Regarding education, patients with a school-level education had a knowledge score of 230 (67.13%), while those with university education scored 523 (70.98%). Among caregivers, school-educated individuals had a score of 134 (46.79%) compared to 453 (58.18%) for those with university education. The Pearson correlation for education is 0.736, signifying a strong positive association; higher education is the most significant factor related to increased knowledge. For residence, rural patients scored 330 (57.13%), while urban patients had a higher score of 480 (67.98%). Among caregivers, those from rural areas scored 214 (36.18%), whereas urban caregivers scored 253 (44.2%). The Pearson correlation coefficient here is 0.423, reflecting a moderate relationship between urban residence and higher diabetes-related knowledge.

In summary, higher income, greater educational attainment, and urban residence are all associated with increased knowledge about diabetes complications in both patients and caregivers. Education stands out as the strongest influencing factor in determining knowledge levels, underscoring the importance of targeted educational interventions to improve diabetes management and complication prevention.

## Discussion

This study evaluated 130 participants - 65 individuals with diabetes and their 65 non-diabetic family caregivers - attending a diabetes specialty clinic at a tertiary care centre. The results showed clear differences in knowledge levels between the two groups, with diabetic patients generally achieving higher scores than their caregivers. Better knowledge scores were significantly associated with higher educational attainment and urban residence, underscoring the impact of socio-demographic factors on understanding and awareness of diabetes management.

Overall, the findings point to substantial gaps in knowledge regarding diabetes management and its complications among both patients and their non-diabetic caregivers. Both patients and caregivers showed limited awareness of neuropathic symptoms such as paresthesia, indicating a significant knowledge gap regarding peripheral neurological complications of diabetes. Since early recognition of these symptoms is crucial for preventing severe outcomes, targeted education for both groups is essential to enhance understanding and promote timely management. Despite the crucial role that education plays in diabetes care [13], both patients and caregivers demonstrated insufficient understanding in several key areas, indicating a need for enhanced educational interventions.

Knowledge about hypoglycemic symptoms and plasma glucose levels was relatively low among both patients and caregivers (Figure 2) [14]. This knowledge gap may stem from several factors, including lack of proper education, inconsistent access to diabetes information resources, and limited structured support for both patients and caregivers in managing diabetes effectively. This is concerning because recognizing and managing hypoglycaemia is critical for preventing severe health events [15]. Educational programs should place greater emphasis on these aspects to ensure that both patients and caregivers can identify and respond appropriately to hypoglycaemic episodes. Both patients and caregivers demonstrated limited knowledge about diabetic complications [16], including chronic co-morbidities, foot care, and peripheral neurological problems. This lack of awareness can lead to delayed identification and treatment of complications, resulting in worse health outcomes [17]. Educational initiatives should focus on providing detailed information about the various complications associated with diabetes and the importance of regular monitoring and preventive care.

A high percentage of both patients and caregivers expressed apprehension about diabetic complications. The high awareness of diabetes related mortality among participants may have dual effects - while it could cause anxiety or fear, it might also motivate better adherence and lifestyle modification. This concern indicates an awareness of the potential severity of diabetes but also highlights a gap between awareness and actionable knowledge. Addressing this gap through comprehensive education can empower patients and caregivers to take proactive steps in managing the disease and preventing complications.

While patients showed a reasonable understanding of diet and exercise, caregivers' knowledge in these areas was lacking [18]. Given that caregivers often help manage daily routines, improving their knowledge about diet and exercise is essential. Health promotion strategies should include practical advice and resources that caregivers can use to support patients in maintaining healthy lifestyles. The study revealed that diabetic patients generally had higher knowledge scores [19] compared to their caregivers. This disparity underscores the importance of including caregivers in educational programs, as their support is vital for effective diabetes management. Higher educational status was significantly associated with better knowledge scores (Table 2) [20], suggesting that educational interventions should be tailored to different educational levels to maximize their effectiveness. Additionally, urban residents had better knowledge compared to rural residents [21], highlighting the need for targeted outreach and education in rural areas.

Patient knowledge about diabetes and its management is fundamental in preventing complications [22]. Empowering patients with the information and skills they need to manage their condition leads to better health outcomes, improved quality of life, and a reduction in the risk of severe, long-term complications.

Caregivers play a crucial role in the management and prevention of complications in people with diabetes. Their knowledge and understanding of diabetes care can significantly impact the patient's health outcomes. Caregivers who are knowledgeable can assist patients in regular blood sugar checks, interpret the results, and help in taking appropriate actions, such as adjusting insulin doses or food intake [23]. They can help in ensuring adherence to medication schedules and recognizing signs of adverse reactions [24]. Diabetic patients are at risk of developing foot ulcers and infections, which can lead to severe complications, including amputations. Knowledgeable caregivers can help with daily foot inspections, ensuring proper hygiene, and recognizing early signs of foot problems [25]. Both low and high blood sugar levels can lead to emergencies if not addressed promptly. Diabetic emergencies, such as diabetic ketoacidosis (DKA) or severe hypoglycaemia, can be life-threatening if not managed promptly [26].

The results of this study suggest that health promotion strategies need to be multifaceted and inclusive, targeting both diabetic patients and their caregivers. Educational programs should be designed to be accessible to individuals with varying levels of education and should address specific knowledge gaps identified in this study. Strategies should also consider the differences between urban and rural populations, ensuring that resources are available and relevant to all demographics. Ongoing education about diabetes and its complications is crucial as the condition and its management may evolve. A caregiver’s knowledge of diabetes care is integral to preventing complications, promoting better health outcomes, and improving the overall quality of life for the patient.

The high level of apprehension about diabetic complications among both groups underscores the importance of comprehensive diabetes education programs. These results highlight critical gaps in knowledge and the potential impact of socio-demographic factors on diabetes management and awareness. The findings will be instrumental in designing effective health promotion strategies to enhance diabetes care and prevent complications.

Limitations

This study has several limitations that may affect the interpretation and generalizability of the findings. Variability in participants' pre-existing knowledge and uncontrolled differences in healthcare access could have influenced the results. The sample was limited in diversity and size, drawn from a single geographic region, and relied on self-reported data, which may introduce bias. Also limited socioeconomic diversity of participants may have influenced the observed associations between income, education and knowledge levels. Additionally, the lack of random sampling and variations in educational program delivery may limit the applicability of these findings to other settings.

## Conclusions

This study highlights significant knowledge gaps regarding diabetes management and its complications among both diabetic patients and their non-diabetic family caregivers, with patients generally demonstrating higher awareness. While areas such as diet and exercise were relatively well understood, critical domains including regular blood sugar monitoring, medication adherence, and recognition of complications - particularly hypoglycaemia, stroke, renal failure, and poor wound healing - showed substantial deficiencies, especially among caregivers. Socio-demographic factors, notably higher education, urban residence, and income, were positively associated with better knowledge, underscoring the influence of social determinants on health literacy.

The findings emphasize an urgent need for targeted, inclusive, and practical educational interventions that actively involve both patients and caregivers. Tailoring these programs to overcome educational and geographical barriers, particularly in rural and lower-education groups, could enhance disease management, improve quality of life, and reduce the risk of severe complications. Addressing the identified knowledge gaps through sustained, community-oriented health promotion strategies will be vital for advancing comprehensive diabetes care. Further studies could evaluate the effectiveness of structured educational interventions for caregivers to enhance their knowledge, self-care support and impact on patient management outcomes.
